# 

**Published:** 1882-10

**Authors:** 


Whole Number, CCCCLIII. | October, 1882. | Number 4, Vol. XLV.

THE

### CHICAGO MEDICAL Journal and Examiner

**EDITORS:**

N. S. DAVIS, M.D., LL.D. JAS. NEVINS HYDE, A.M., M.D.

DANIEL R. BROWER, M.D.

Chicago Medical Press Association.

Aug. 2D

1875.

CHICAGO:

*PUBLISHED BY THE CHICAGO MEDICAL PRESS ASSOCIATION,*

188 Clark Street.

☞ Read the Notice of this Journal among Advertisements

